# First-in-Man Use of Intraoperative Electrophysiological Mapping to Evaluate the Efficacy of the EnCompass Clamp During a Cox-IV Maze Procedure

**DOI:** 10.7759/cureus.66131

**Published:** 2024-08-04

**Authors:** Zain Khalpey, Usman Aslam, Ujjawal Kumar, Leslie Epting

**Affiliations:** 1 Department of Cardiothoracic Surgery, HonorHealth, Scottsdale, USA; 2 Department of General Surgery, HonorHealth, Phoenix, USA; 3 School of Clinical Medicine, University of Cambridge, Cambridge, GBR; 4 Department of Electrophysiology, Abbott Inc., Chicago, USA

**Keywords:** tricuspid valve repair, mitral valve repair, electrophysiological mapping, surgical ablation, atrial fibrillation

## Abstract

This case report describes the first-in-man use of intraoperative electrophysiological (EP) mapping to evaluate the efficacy of the EnCompass clamp (AtriCure, Inc., Mason, OH) during a Cox-IV Maze procedure. A 53-year-old male with paroxysmal atrial fibrillation and severe mitral valve regurgitation underwent mitral valve repair with concomitant surgical ablation for atrial fibrillation. Intraoperative 3D EP mapping was performed using the Abbott EnSite Precision system (Abbott Inc., Chicago, IL) before ablation, after initial radiofrequency ablation with the AtriCure EnCompass clamp, and after the full Cox-IV Maze procedure was completed.

The pre-ablation map showed approximately 80-85% high voltage areas in the posterior left atrial wall. Initial ablation with the EnCompass clamp reduced high voltage areas to 30-35%. The final map following the Cox-IV Maze procedure demonstrated near-complete electrical silence, with only 5-10% of the atrial surface retaining high voltage activity. This represents an estimated 88% reduction in high-voltage areas from baseline. The patient had an uncomplicated postoperative course apart from one episode of postoperative atrial fibrillation requiring direct current (DC) cardioversion.

This case demonstrates the utility of intraoperative EP mapping in guiding and confirming the efficacy of surgical ablation procedures, as well as the effectiveness of combining the EnCompass clamp with a full Cox-IV Maze in achieving comprehensive atrial electrical isolation. The EnCompass clamp can be used for ablations with a beating heart, thus reducing the aortic cross-clamp time and therefore minimizing the total myocardial ischemia time.

## Introduction

Atrial fibrillation (AF) is the most common arrhythmia and leads to a significant morbidity and mortality burden, particularly in patients older than 65 years of age [1]. It is characterized by irregular and often rapid heart rates and can pose significant challenges during cardiac surgeries, particularly those involving valve repairs or replacements. In patients undergoing concomitant mitral and tricuspid valve repairs, the presence of preoperative paroxysmal AF can complicate the surgical procedure and increase the risk of postoperative complications if left untreated. Concomitant surgical management of AF alongside other cardiac surgical procedures is therefore a class 1 recommendation as per the Society of Thoracic Surgeons and the Heart Rhythm Society guidelines [2,3]. Despite these clear benefits, concomitant surgical ablation is underperformed in patients with AF undergoing cardiac surgery. A large national study of patients with AF who were undergoing elective cardiac surgery (n = 86,941) found that less than half underwent concomitant surgical ablation for their AF, indicating a low uptake of this concomitant treatment approach [4], which would typically be performed using a device such as the Isolator Synergy Bipolar radiofrequency clamp (AtriCure Inc., Mason, OH) [5].

Treating paroxysmal AF in these patients is crucial for several reasons. Firstly, paroxysmal AF can increase the risk of complications during and after cardiac surgery, such as stroke, bleeding, and heart failure exacerbation. Restoring sinus rhythm can reduce these risks and improve overall surgical outcomes. Secondly, AF is associated with an increased risk of thrombus formation, which can lead to stroke or other thromboembolic events. Treating AF can reduce this risk and improve long-term patient outcomes. Thirdly, patients with untreated AF have a lower quality of life due to the persistence of arrhythmia-related symptoms and reduced exercise tolerance. Treating AF can alleviate these symptoms and improve overall well-being. Lastly, untreated AF can lead to long-term complications, such as tachycardia-induced cardiomyopathy, heart failure, and cognitive impairment. Addressing AF during valve repair can prevent or mitigate these complications.

The most effective approach for concomitant AF treatment is represented by the bilateral Cox-Maze operation. This method replaces the traditional “cut-and-sew” technique for lesion sets with ablation lines. The Cox-IV Maze (CM-IV) technique improves outcomes for surgical ablation by reducing operative times and complications. However, the CM-IV procedure can still significantly increase the aortic cross-clamp time and therefore increase the duration of myocardial ischemia.

To address this issue, the Isolator Synergy EnCompass clamp (AtriCure, Inc., Mason, OH) has emerged as a promising treatment option. The EnCompass clamp is a radiofrequency ablation (RFA) instrument designed to create continuous, transmural lesions in the atrial tissue, effectively isolating the pulmonary veins and other critical areas responsible for initiating and perpetuating AF [6]. By isolating these areas, the clamp aims to restore normal sinus rhythm and prevent the recurrence of AF. The EnCompass clamp is strategically positioned around the pulmonary veins and other targeted areas of the atria. The clamp's unique design allows for the precise application of radiofrequency energy, creating continuous lesions that effectively block the abnormal electrical signals responsible for AF [7]. This approach eliminates the need for atriotomy and cross-clamping and reduces the risk of complications associated with traditional open-heart procedures. This clamp enables the full “box lesion” set to be made with just one application, simplifying the process of surgical ablation and eliminating the need for atriotomy for ablation and therefore minimizing aortic cross-clamp time and myocardial ischemia. This is especially useful in low EF patients undergoing CABG with hostile aorta unsuitable for a cross-clamp. It has also been shown to create more reliable transmural lesions than a reference system, as well as creating wider lesions with greater tissue volumes ablated and energy delivered [8].

The use of the EnCompass clamp for RFA in conjunction with mitral and tricuspid valve repairs offers several potential benefits, including improved long-term outcomes, reduced risk of stroke and other thromboembolic events, and enhanced quality of life for patients. RFA using the EnCompass clamp, combined with a surgical CM-IV ablation has the added benefit of achieving complete electrophysiological silence. This comprehensive surgical treatment of AF during valve repair procedures should reduce/eliminate the AF burden, eliminating the need for subsequent interventions or long-term anticoagulation therapy. In this case report, we describe the successful use of the EnCompass clamp as well as intra-operative 3D electrophysiological mapping before and after concomitant surgical ablation.

## Case presentation

**Figure 1:**
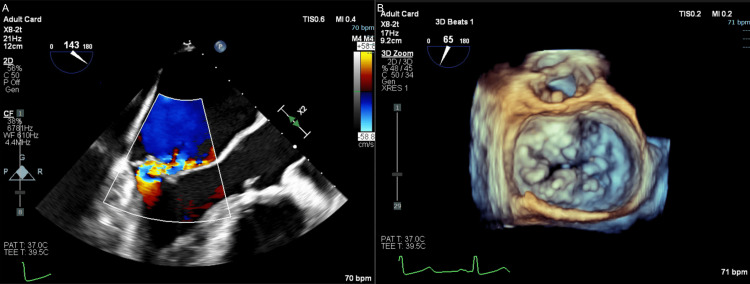
Preoperative transesophageal echocardiography (TEE).

A 53-year-old male was referred for cardiothoracic surgical evaluation of mitral valve prolapse and consideration for mitral valve surgery. His chief complaint was occasional shortness of breath and lightheadedness. Echocardiography showed severe mitral valve regurgitation with a Barlow’s valve, prolapse, and flail posterior leaflet (Figure 1). Mild tricuspid regurgitation and severe left atrial enlargement were also noted, with a left ventricular ejection fraction of 55% and grade III left ventricular diastolic dysfunction. Myocardial perfusion imaging showed no regions of ischemia. His past medical history was significant for paroxysmal AF (on apixaban for thromboembolism prophylaxis), hypertension, and obesity with a BMI of 37 kg/m^2^. He consented to and was subsequently scheduled for mitral valve surgery with tricuspid valve repair and concomitant surgical ablation of AF.

**Figure 2:**
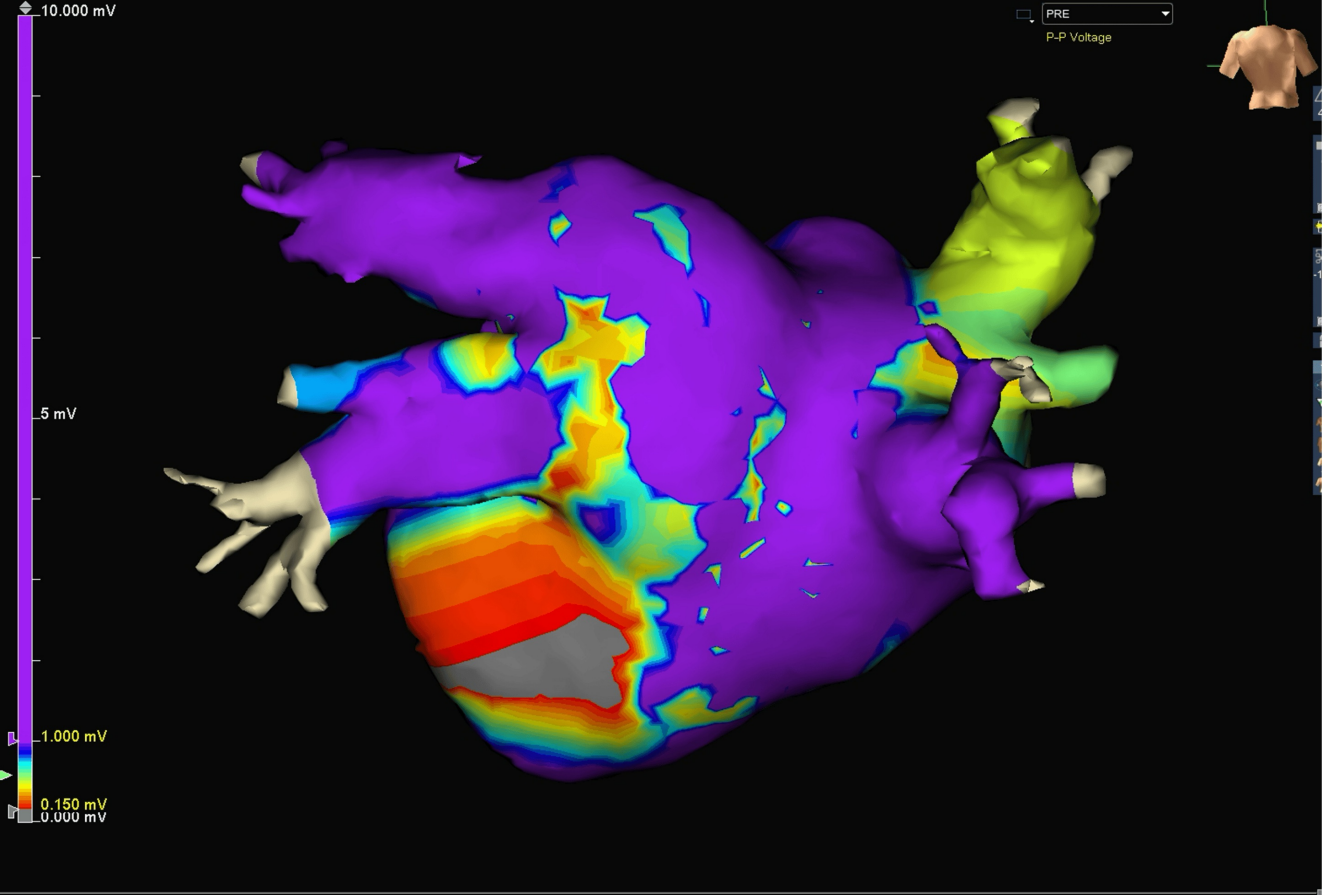
Pre-ablation baseline voltage map of the left atrium and pulmonary veins. This image shows baseline electrophysiological (EP) activity on a beating heart, focusing on the left atrial wall and pulmonary veins before ablation. The color-coded voltage map indicates that approximately 80-85% of the posterior wall exhibits high voltage (purple), representing healthy atrial tissue. The voltage scale ranges from 0.000 mV (gray) to 10.000 mV (purple), with intermediate voltages shown in red, yellow, green, and blue. Lower voltage areas are visible in the central and inferior portions, potentially indicating natural anatomical variations or pre-existing low-voltage regions. This pre-ablation map serves as a crucial reference for assessing the effectiveness of subsequent ablation procedures in creating areas of electrical isolation.

Following a full median sternotomy, the ascending aorta and right femoral vein were cannulated (20 Fr and percutaneous 25 Fr cannulae, respectively) and cardiopulmonary bypass was initiated. Before starting the process of ablation, complete electrophysiological mapping was performed using the EnSite Precision mapping system (Abbott Inc., Chicago, IL). A 3D map was created by moving the mapping probe over the left pulmonary vein, left posterior atrial wall, roof of the left atrium, and right pulmonary vein. Tissue with electrical activity greater than 1.0 mV was considered healthy or viable and represented with a purple color on the density map. Tissue with less than 0.15 mV of activity was considered scarred, ablated, or diseased and represented with a grey color. The pre-procedure voltage map obtained 4,476 points, of which only 946 points were used for the graphic (Figure 2). Many points were either duplicates in the same location or close in proximity to the geometric surface measured. The mapping process took around three minutes in total.

**Figure 3:**
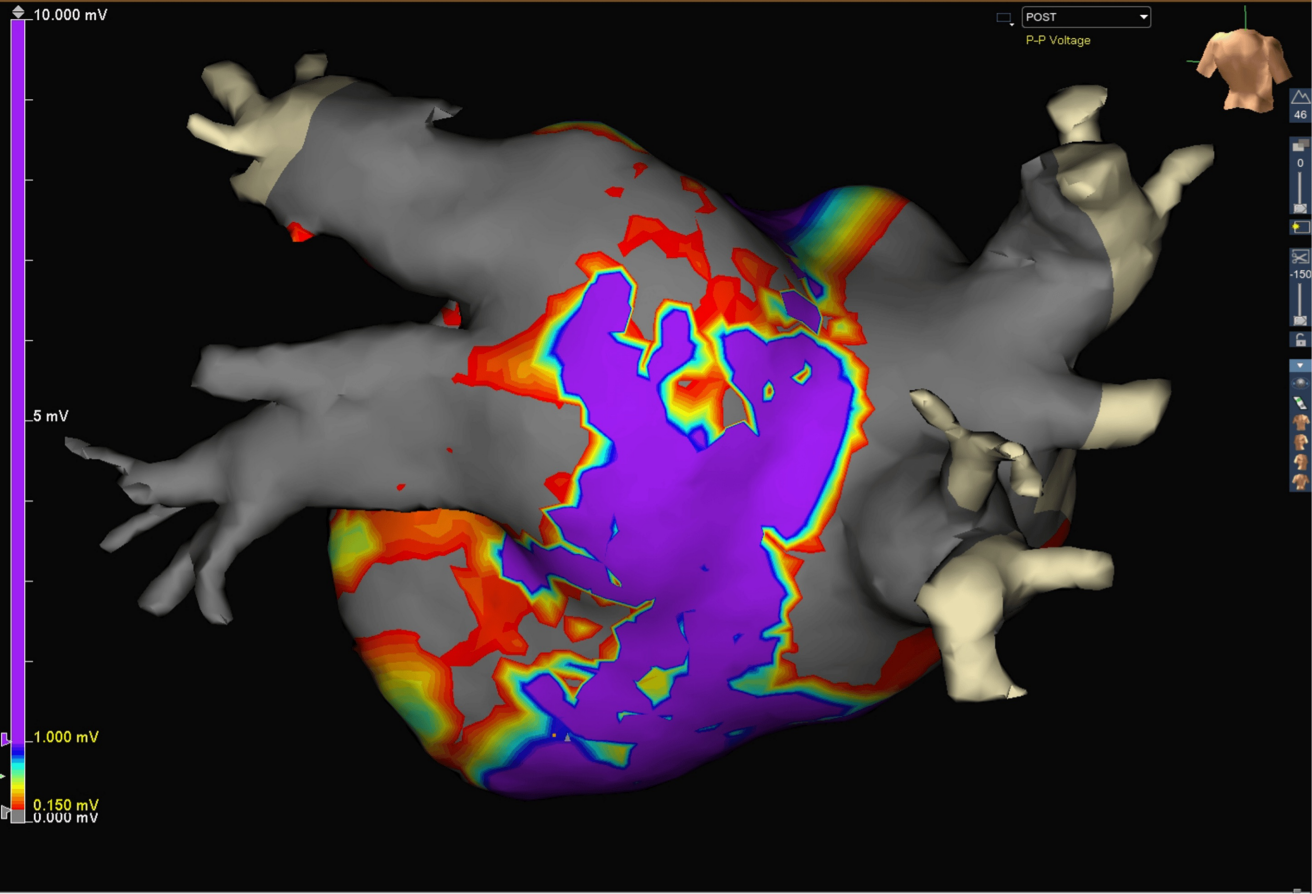
Post-ablation voltage map: partial silencing of posterior left atrial wall and pulmonary vein. This voltage map demonstrates the effects of EnCompass clamp ablation on the posterior left atrial wall and pulmonary veins. Post-procedure, approximately 30-35% of the posterior wall retains high voltage activity (purple), while significant low voltage areas (gray) are now present. This indicates partial electrical silencing of the target regions. Some residual electrical activity persists in the posterior wall, suggesting areas that may require further treatment. The image provides a quantitative assessment of ablation efficacy, showing a substantial reduction in high-voltage regions compared to the pre-ablation state.

Once the mapping was completed, we proceeded with the left atrial box lesion of the CM-IV procedure using the Isolator Synergy EnCompass device. This is a dual-electrode bipolar radiofrequency non-irrigated clamp with a 105 mm electrode length for cardiac tissue ablation. A magnetic-tipped red rubber loop is used to position the clamp around the posterior left atrium through the oblique and transverse cardiac sinuses to create the left atrial lesion box set. The posterior left atrium and right atrium were isolated with transmural lesions made using the EnCompass clamp. The mapping grid was used to create a post-ablation electrophysiological map and we observed complete isolation of both the left and right atria (Figure 3). A 40 mm left atrial appendage clip (AtriClip, AtriCure Inc., Mason, OH) was applied at this point for thromboembolic prophylaxis.

At this point, the aortic cross-clamp was applied, and the right atrium was then opened to proceed with the tricuspid valve repair using a modified DeVega stitch approach. The tricuspid valve area was measured using a 20 mL syringe in the orifice of the tricuspid valve, to provide a 2 cm^2^ area for placement of the DeVega stitch and prevent excess strain on the tricuspid valve annuloplasty repair. A 3-0 pledgeted polypropylene suture was taken in parallel around the annulus of the tricuspid valve over the posterior leaflet (from the anteroposterior commissure to the posteroseptal commissure) and plicated. This obliterates the posterior tricuspid leaflet, achieving bicuspidization of the valve, as pioneered by Cohn [9]. While this was tested, the valve was competent, and attention was turned to repairing the mitral valve using a transseptal approach.

**Figure 4:**
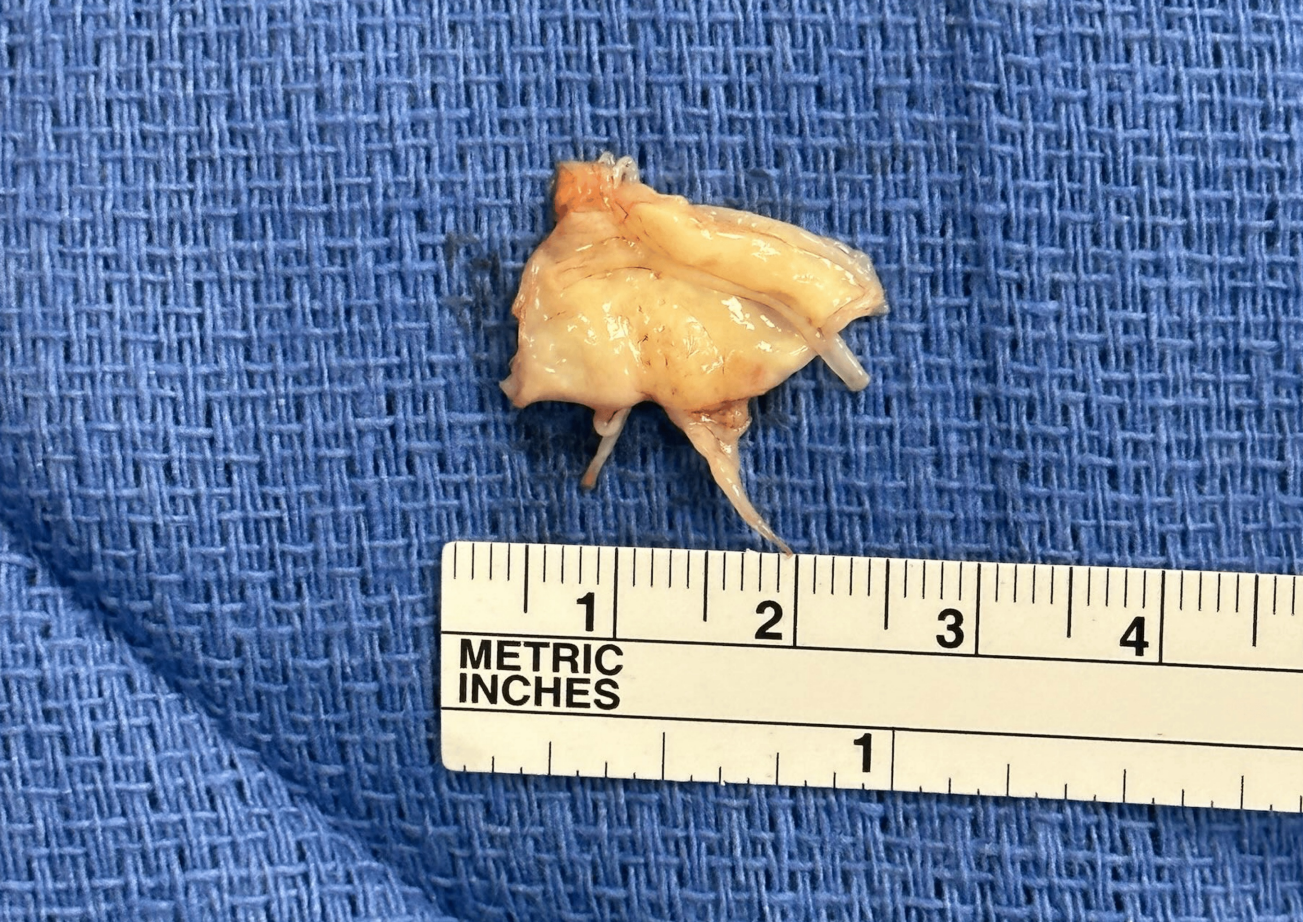
Resected P2/P3 wedge of the mitral valve.

The mitral valve leaflets were inspected, finding torn P2 and P3 chords. A P2/P3 wedge was resected (Figure 4) and a valvuloplasty was performed. The two cut edges were joined with a running 5-0 polypropylene C-1 suture, restoring the leaflet heights to normal. Neochord was utilized for the P1 and P2 sections of the valve down to the medial papillary muscle head. Non-pledgeted parallel 2-0 Ethibond sutures (Ethicon Inc., Cincinnati, OH) were placed at the commissure through the ring and weaved around the circumference of the annulus, then sequentially through the sewing ring of the 36 mm mitral valve Physio II annuloplasty ring (Edwards Lifesciences, Irvine, CA). The valve ring was then seated and the subvalvular space was inspected and tested carefully. The trans-septal incision was then closed with a running 4-0 polypropylene suture.

Next, the remainder of the CM-IV procedure was performed. An atriotomy was made in Sondergaard's groove and a full four-lesion set was performed to include the coronary sinus and mitral isthmus lesion sets, though the lateral mitral line (from the P1 segment to the base of the left atrial appendage) was not abated. These lesions were made using the cryoablation probe, before proceeding with the right atrial lesion set. This was completed using mainly RFA with cryoablation used for the lesion extending from the right atrial free wall down to the tricuspid valve annulus. The total cardiopulmonary bypass time was 185 minutes and the aortic cross-clamp time was 96 minutes.

**Figure 5:**
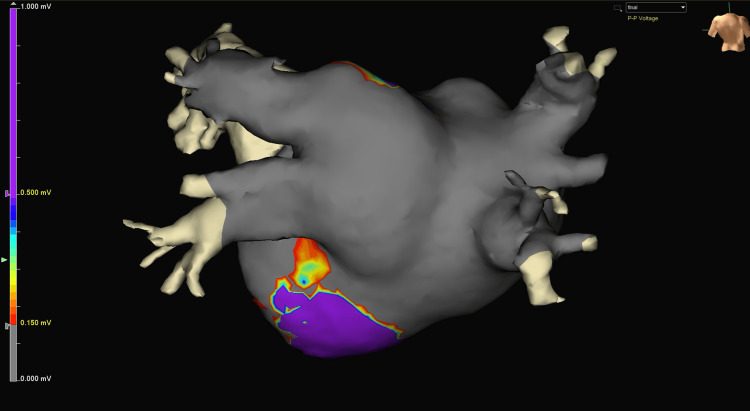
Completion of Cox-IV Maze mapping on a beating heart. The patient was in normal sinus rhythm. This 3D voltage map depicts the posterior wall of the beating heart in normal sinus rhythm following the completion of the Cox-IV Maze procedure. The posterior wall is now almost completely electrically silent, with approximately 90-95% of the visible surface now showing low voltage, and only 5-10% of the area retaining high voltage. This map demonstrates a 71% further reduction in high-voltage areas compared to the previous post-ablation map (after the EnCompass clamp ablations alone) and an 88% overall reduction from the pre-ablation state. These findings indicate substantial improvement in electrical isolation, suggesting highly effective ablation, with the posterior wall now almost completely electrically silent.

We then created a post-procedure electrophysiological map and observed near-complete electrical silence of the left and right atria (Figure 5). In the 3D maps, 6,072 points were collected on the post-procedure voltage map with 1,167 points used for the graphic. Notably, the post-procedure map at the superior posterior wall near the roof appears electrically isolated from the bulk of the left atrial chamber. Infrequent and random voltage signals are noted in this area, with the patient being in normal sinus rhythm. A final transesophageal echocardiography (TEE) confirmed satisfactory repair of both the mitral and tricuspid valves, with no more flow reversal in pulmonary veins or hepatic veins seen. The chest was closed in our usual institutional manner, with the patient being discharged to the ICU in a hemodynamically stable state.

Rhythm monitoring was performed postoperatively using standard 24-hour telemetry monitoring and daily 12-lead EKGs. Postoperative AF was defined by the Society of Thoracic Surgeons' definition as an episode of AF lasting longer than one hour or less than an hour and requiring medical or surgical intervention. The patient had an episode of stable, asymptomatic postoperative AF and was started on amiodarone on the fourth postoperative day. Following discontinuation, he had an episode of asymptomatic low-voltage atrial flutter, requiring direct current (DC) cardioversion. He had an otherwise uncomplicated postoperative course and was successfully discharged home on the fifth postoperative day and remains off antiarrhythmic medications to this day. At one-, three-, six-, and 12-month outpatient follow-up visits, the patient had experienced complete symptom resolution, with no episodes of AF. Both his antiarrhythmic medication and anticoagulant had been safely discontinued.

## Discussion

This case report demonstrates the successful use of the AtriCure EnCompass clamp in conjunction with intraoperative 3D electrophysiological (EP) mapping during a CM-IV procedure for a patient with paroxysmal AF as well as severe mitral and tricuspid valve regurgitation. The pre-ablation EP map showed approximately 80-85% high voltage areas in the posterior left atrial wall. Initial ablation with the EnCompass clamp reduced high voltage areas to 30-35%, while the final map following the complete CM-IV procedure demonstrated near-complete electrical silence, with only 5-10% of the atrial surface retaining high voltage activity. This represents an estimated 88% reduction in high-voltage areas from baseline.

Effectiveness of the EnCompass clamp

The EnCompass clamp, introduced by AtriCure in April 2022, represents a significant advancement in surgical AF treatment. Its ability to create simultaneous transmural lesions of the four "box lesion" sides with a single application improves the effectiveness and efficiency of surgical ablation. In this case, the EnCompass clamp demonstrated its efficacy by significantly reducing high voltage areas in the atrial tissue after initial application.

The EnCompass clamp offers several advantages over traditional ablation techniques. Firstly, it reduces procedural time through its single-application approach for the box lesion set, potentially decreasing the overall duration of the ablation procedure, specifically the aortic cross-clamp time, and therefore the duration of myocardial ischemia. Secondly, the clamp's design ensures consistent contact and energy delivery, potentially leading to more reliable transmural lesions, thus improving transmurality [8]. Lastly, the EnCompass clamp offers flexibility in its application. While it can be used for non-atriotomy ablation, it can also be incorporated into procedures requiring atriotomy, as demonstrated in this case for mitral valve repair. In cases where an atriotomy is not required, the EnCompass clamp is perfect, as it provides a rapid option for a posterior encircling lesion including the posterior wall and pulmonary veins in one application [10]. This allows the lesions to be made with the heart beating and without requiring the application of the aortic cross-clamp, reducing myocardial ischemia.

Utility of intraoperative EP mapping

The Abbott EnSite Precision cardiac mapping system demonstrates the value of intraoperative EP mapping in guiding and confirming surgical ablation efficacy. It offers real-time feedback for immediate assessment and adjustment of ablation lesions, high-resolution mapping for precise identification of areas needing further ablation, and comprehensive evaluation through pre-, interim, and post-procedure mapping. This allows surgeons to track electrical isolation progress throughout the procedure, enhancing the precision and effectiveness of ablations and potentially improving overall outcomes. Through intraoperative EP mapping, we knew that there was an estimated 88% reduction in activity in high-voltage areas, conferring significant confidence that the patient’s AF would be successfully treated. If certain areas of high voltage had remained, this information would have allowed us to go back and make additional lesions to ensure complete electrical silence, maximizing chances of procedural success and symptom resolution.

The EnSite Precision system uses impedance fields generated by surface electrodes. An 8 kHz signal is sent alternately through each pair of electrodes to create a voltage gradient along each axis, forming a transthoracic electrical field [11]. This allows for accurate three-dimensional positioning of catheter electrodes and precise voltage mapping. Compared to other intraoperative mapping methods, such as direct contact mapping or fluoroscopy-guided techniques, the EnSite system offers superior spatial resolution and the ability to create detailed 3D maps of cardiac electrical activity.

Clinical implications

Concomitant surgical ablation of AF during cardiac procedures maximizes benefits by restoring stable sinus rhythm, improving survival, and reducing stroke incidence. AF is driven by re-entrant circuits involving key atrial structures, which the CM-IV procedure interrupts through strategic ablation lesions in both atria [12]. For mitral valve surgery patients with AF, a comprehensive left-sided lesion set is crucial, with right-sided ablation further reducing recurrence risk [13].

The combination of the EnCompass clamp and intraoperative EP mapping demonstrates potential benefits for patients undergoing concomitant surgical ablation. It offers improved efficacy through a significant reduction in high-voltage areas, a tailored approach via real-time EP mapping for personalized ablation strategies, and reduced procedural risks due to the EnCompass clamp's efficiency, potentially leading to shorter procedural and aortic cross-clamp times.

Patient outcomes

The patient in this case experienced a generally uncomplicated postoperative course, with only one episode of postoperative AF (medically managed) and an episode of atrial flutter requiring cardioversion. It is possible that had the lateral mitral line been ablated, the flutter would not have developed, and this is something that has subsequently been modified in our institutional clinical practice. At follow-up visits up to 12 months post-procedure, the patient remained in sinus rhythm without antiarrhythmic medications, demonstrating complete symptom resolution. This outcome is encouraging and suggests that the combined use of the EnCompass clamp and intraoperative EP mapping may contribute to improved long-term success rates in AF treatment.

Future research should focus on larger, prospective studies comparing outcomes of patients treated with the EnCompass clamp and intraoperative EP mapping to those treated with traditional techniques such as the traditional CM-IV procedure undertaken concomitantly with atrioventricular valve surgery. Additionally, long-term follow-up studies are needed to assess the durability of ablation lesions created using this approach.

## Conclusions

The EnCompass clamp simplifies the delivery of transmural box lesions across the pulmonary vein ostia in just one application, potentially reducing procedural time and myocardial ischemia. Intraoperative 3D cardiac electrophysiological mapping using the EnSite Precision cardiac mapping system proves to be a reliable method to confirm electrical silence after pre-atriotomy ablation sets using the EnCompass clamp and after bi-atrial CM-IV ablation. This case report demonstrates the first described safe application of the EnCompass clamp with concomitant CM-IV surgical ablation to achieve near-complete electrical silence, as evidenced by an 88% reduction in high-voltage areas. The combination of these techniques offers the potential for improved efficacy, tailored approach, and reduced procedural risks in treating AF during cardiac surgery. Long-term follow-up and larger studies are needed to further validate these promising results.
